# Simultaneous Massive Esophageal Mucosal Candidiasis and Profound Cytomegaloviral Esophageal Ulcers with Recurrence of Both Infections 12 Years Later in a Patient with Long-Standing AIDS: Endoscopic, Radiologic, and Pathologic Findings

**DOI:** 10.1155/2022/9956650

**Published:** 2022-02-27

**Authors:** Inayat Gill, Ahmed Edhi, Mitual Amin, Mitchell S. Cappell

**Affiliations:** ^1^Division of Gastroenterology, Department of Internal Medicine, William Beaumont Hospital at Royal Oak, 3535 W. Thirteen Mile Rd, Royal Oak, MI 48073, USA; ^2^Department of Pathology, William Beaumont Hospital at Royal Oak, 3600 W. Thirteen Mile Rd, Royal Oak, MI 48073, USA; ^3^Department of Pathology, Oakland University William Beaumont School of Medicine, 3600 W. Thirteen Mile Rd, Royal Oak, MI 48073, USA; ^4^Division of Gastroenterology, Department of Medicine, Building 1, Room 3210, Aleda E. Lutz VA Medical Center, 1500 Weiss St, Saginaw, MI 48602, USA

## Abstract

Immunocompromised patients with acquired immunodeficiency syndrome (AIDS) can develop opportunistic esophageal candidial and cytomegaloviral infections. A case is reported which extends the clinico-endoscopic severity of these infections. A 32-year-old bisexual man with AIDS since 1997, and intermittently compliant with antiretroviral therapy, presented (2007) with dysphagia and 32 kg-weight loss. EGD revealed a massive, cheesy, esophageal mucosal exudate from *Candida albicans*. Cytomegalovirus was isolated by viral culture. The patient improved after fluconazole/ganciclovir therapy. The patient re-presented (2019) with hematemesis and dysphagia. EGD revealed cheesy esophageal exudate and profound “punched out” esophageal ulcers mimicking pseudo-diverticula. Histopathology confirmed candidiasis. Viral cultures revealed cytomegalovirus. Barium esophagram revealed deep esophageal ulcers/pseudo-diverticula. Repeat EGD 8 weeks later after ganciclovir/micafungin therapy revealed mostly healed lesions. This demonstrates that AIDS patients may have massive mucosal esophageal candidiasis; that both infections can recur years after apparent eradication; and that cytomegaloviral esophageal ulcers may be profound and mimic pseudo-diverticula. A comprehensive literature review revealed only one abstract of esophageal pseudo-diverticula associated with cytomegalovirus. Simultaneous esophageal candidial and CMV infections have also been rarely reported in immunocompromised patients without AIDS.

## 1. Introduction

Immunocompromised patients with acquired immunodeficiency syndrome (AIDS) can develop opportunistic esophageal candidial and cytomegaloviral (CMV) infections. A case is reported which extends the spectrum of clinical, endoscopic, radiologic, and pathologic severity of these infections associated with AIDS by reporting (1)-simultaneous massive esophageal mucosal candidiasis and CMV; (2)-recurrence of both infections 12 years later; and (3)-occurrence of CMV “punched out” ulcers so deep that they mimicked esophageal “pseudo-diverticula” at esophagogastroduodenoscopy (EGD) and barium esophagram. These phenomena are attributed to the patient's poor adherence to antiretroviral therapy.

## 2. Case Presentation

**Figure 1:**
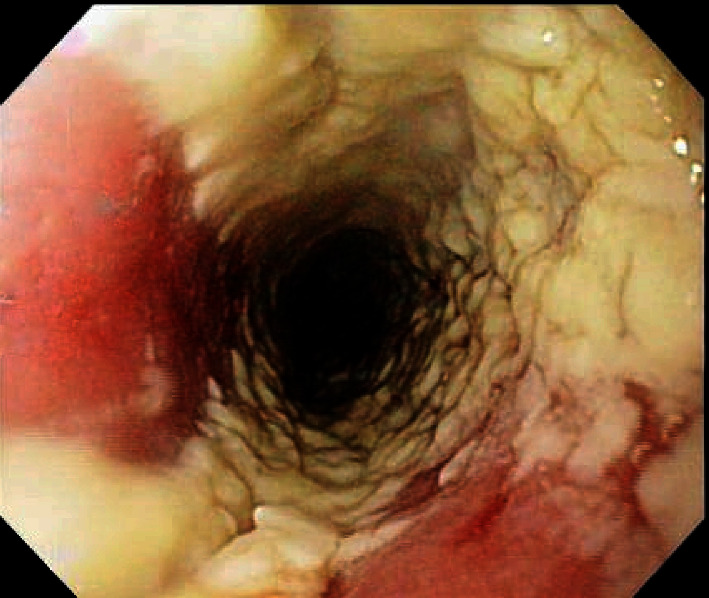
Esophagogastroduodenoscopy (EGD) performed in 2007 in a patient with acquired immunodeficiency syndrome (AIDS), carried out for the indications of severe odynophagia, dysphagia, and weight loss revealed massive, cheesy, tightly adherent, esophageal mucosal exudate/esophagitis from candidiasis (see Figure 2). Even though the esophageal candidiasis was massive in 2007, it was very superficial with superficial mucosal ulcers, as opposed to the deep “punched out” ulcers reported in 2019 (Figure 5), attributed to esophageal cytomegaloviral infection.

**Figure 2:**
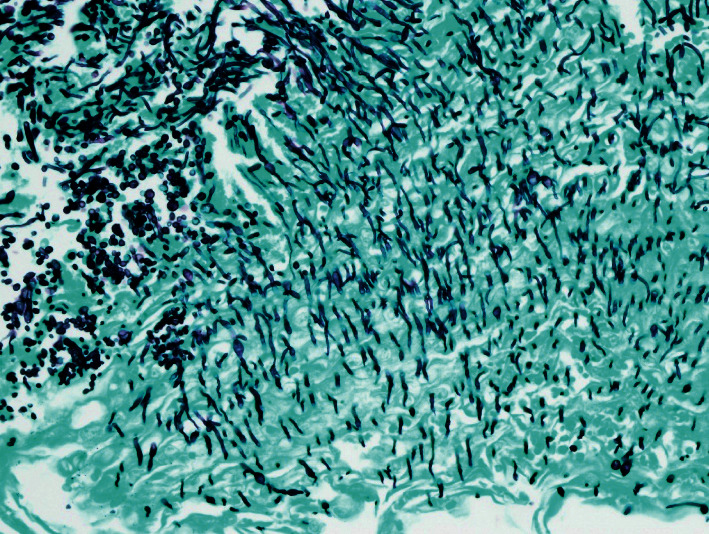
Gomori methenamine silver (GMS) stain of endoscopic biopsies of cheesy esophageal exudate demonstrates black spherical yeast forms (upper left) and elongated hyphae (throughout photomicrograph), with the characteristic morphology of *Candida*.

A 32-year-old Black American bisexual man with AIDS since 1997, prior *Pneumocystis carinii* pneumonia, and intermittent compliance with antiretroviral therapy, presented (2007) with chronic odynophagia, dysphagia, and 32 kg-involuntary-weight loss, without prior administration of esophagotoxic medications, corticosteroids, illicit drugs, alcoholism, diabetes mellitus, or gastroesophageal reflux. The patient was relatively indigent with only intermittent employment. Physical examination revealed a temperature of 38.1°C, temporal and intercostal muscle wasting, thrush, and a nontender abdomen. Hemoglobin was 10.1 g/dL (normal: 13.5–17 g/dL), leukocytes were 4.8 bil/L (normal: 3.5–10.1 bil/L), and CD4 cells were 68 mil/L (normal: 433–1722 mil/L). EGD revealed massive, cheesy, adherent, and esophageal mucosal exudate/esophagitis (Kodsi endoscopic candida classification grade 4 [1], Figure 1). *Candida albicans* was isolated from esophageal fungal cultures and demonstrated histologically (Figure 2). Hematoxylin-eosin stain of esophageal biopsies (without immunohistochemistry) revealed insufficiently deep samples to histologically demonstrate CMV, but CMV was isolated by viral culture of esophageal biopsies. The patient improved after intravenous fluconazole and ganciclovir therapy.

The patient re-presented (2019), intermittently compliant with antiretroviral therapy, with acute hematemesis and chronic odynophagia and dysphagia. Physical examination revealed a temperature of 37.9°C, blood pressure of 150/90 mmHg, pulse of 99 beats/minute, orthostasis, BMI = 15.8 kg/m^2^, temporal and intercostal muscle wasting, thrush, and nontender abdomen. Hemoglobin was 6.5 g/dL, leukocytes were 4.4 bil/L, and CD4 cells were 4 mil/L.

**Figure 3:**
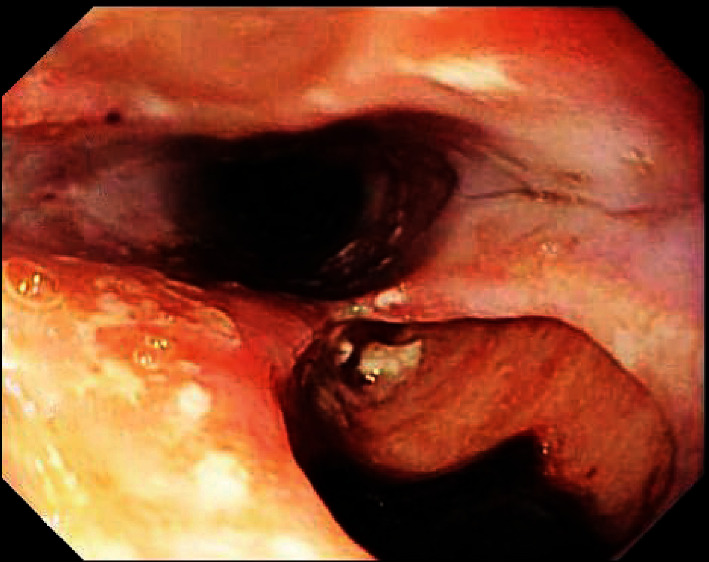
Esophagogastroduodenoscopy performed in 2019 for indications of acute hematemesis and chronic odynophagia and dysphagia reveals profound, irregular, “pseudo-diverticular” esophageal ulcers with a maximal diameter of 12 mm and a depth of 15 mm that are located posteriorly to the normal esophageal lumen: Cytomegalovirus was isolated from viral cultures of endoscopic biopsies taken from these esophageal ulcers.

**Figure 4:**
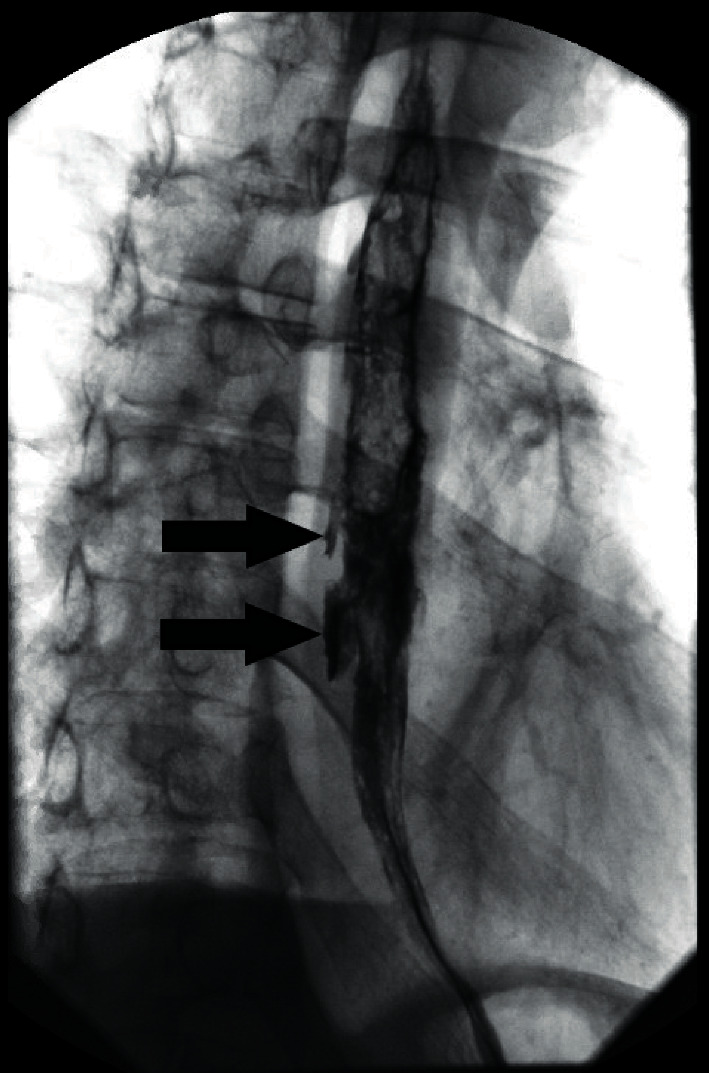
Barium esophagram reveals irregular, posterior, nonperforating, deep esophageal ulcers (arrows) that mimic esophageal pseudo-diverticula in extending posteriorly beyond the normal esophageal wall.

After transfusing 2 units of packed erythrocytes and administering intravenous pantoprazole, EGD was performed, which demonstrated cheesy esophageal exudate and profound esophageal “punched out” ulcers (maximal depth = 15 mm) that mimicked pseudo-diverticula (Figure 3). Ulcers were not treated endoscopically because high-risk stigmata of recent hemorrhage (SRH) were absent. Histopathology of esophageal biopsies confirmed candidiasis (Kodsi endoscopic candida classification grade 3). Polymerase chain reaction and viral culture of esophageal biopsies (taken from ulcer edges and sides) revealed cytomegalovirus (no Herpes simplex). Barium esophagram revealed deep, irregular, posterior, nonperforating, esophageal ulcers, resembling pseudo-diverticula, without esophageal strictures (Figure 4).

## 3. Outcome and Follow-Up

**Figure 5:**
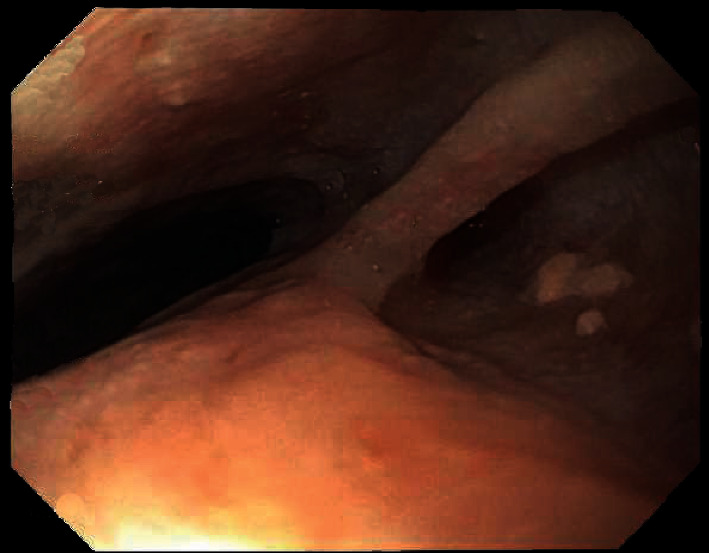
Repeat esophagogastroduodenoscopy (EGD) in 2019, 8 weeks after initiating therapy with ganciclovir and micafungin, for profound esophageal pseudo-diverticular ulcers and severe esophageal candidiasis, revealed mostly healed esophageal cytomegaloviral ulcers and esophageal candidiasis.

After intravenous ganciclovir and micafungin therapy, hemoglobin stabilized at 8.3 g/dl and dysphagia resolved. Repeat EGD 8 weeks later revealed mostly healed esophageal candidiasis and CMV ulcers (Figure 5). He has returned three times to the hospital with recurrent dysphagia during the subsequent 6 months from esophageal candidiasis, each time after self-discontinuing the micafungin therapy.

## 4. Discussion

AIDS patients can develop deep esophageal CMV ulcers [2] and widespread candidiasis [3] from immunocompromise. This work demonstrates that, in immunosuppressed patients, esophageal candidiasis may be massive; that both infections can recur 12 years after apparent eradication; and that CMV esophageal infection may cause so profoundly “punched out” ulcers so as to resemble esophageal pseudo-diverticula.

A comprehensive literature review revealed one prior report of esophageal deep ulcers that resembled esophageal pseudo-diverticula associated with CMV [4]. A 52-year-old male, HIV-seropositive patient with <200 CD4 cells/mm^3^ presented with dysphagia. EGD revealed esophageal pseudo-diverticula/ulcers. Symptoms improved after unspecified treatment. However, this case was only reported as an abstract, and the ulcer biopsies were reported as “consistent,” but not diagnostic, of CMV.

Oropharyngeal or esophageal candidiasis frequently occurs in patients with AIDS, especially with advanced disease. Esophageal mucosal candidiasis and CMV esophageal ulcers can also occur in other immunocompromised patients without AIDS [5–7]. Esophageal mucosal candidiasis occurs in patients undergoing chemotherapy or having hematologic malignancies, including lymphoma and leukemia [5, 6]. CMV is present in immunocompromised patients with solid organ or bone marrow transplants [7]. Simultaneous presentation of candidial and CMV esophageal infections from other causes of immunosuppression, without HIV infection, is rare [8–12]. One patient with immunosuppression secondary to prednisolone and cyclophosphamide therapy for severe interstitial pneumonia developed simultaneous candida and CMV esophageal infections [8]. Also, a kidney transplant recipient receiving methylprednisolone therapy had recurrent aphthous stomatitis secondary to candida and CMV [9]. Two patients with kidney transplants receiving tacrolimus therapy had simultaneous occurrence of oral mucosal ulcers from candida and CMV [10]. A patient with Hodgkin's lymphoma receiving intravenous immunoglobulin had concurrent candidial and CMV infections [11]. A review of the literature revealed that simultaneous candida and CMV infections can also rarely occur in other organs, such as the cornea [12].

Extreme symptoms (dysphagia and profound weight loss), signs (cachexia evidenced by extreme weight loss, very low BMI, and temporal and intercostal muscle wasting), endoscopic findings (massive cheesy esophageal mucosal exudate), radiologic findings (deep ulcers mimicking pseudo-diverticula), pathologic findings, and recurrence of both infections after 12 years are all attributed to profound immunocompromise (last CD4 cell count = 4 mil/L) from poor adherence to antiretroviral therapy. Study limitations include that it is retrospective, consists of one case, and presents only qualitatively new findings representing a severe spectrum of findings for esophageal candidiasis and CMV. This work illustrates the importance of antiretroviral therapy in AIDS patients to prevent opportunistic infections and the inclusion of esophageal CMV in the differential of esophageal pseudo-diverticula in severely immunocompromised patients. *Candida* unlikely played a significant pathophysiologic role in causing the profound “punched out” esophageal ulcers (Figure 5) that resembled esophageal pseudo-diverticula on barium swallow (Figure 4) reported in 2019, and these profound ulcers are most likely attributable to esophageal CMV infection [3].

CMV is diagnosed by immunohistochemical staining with anti-CMV DNA, in situ hybridization with CMV DNA, or isolation of CMV by viral cultures of esophageal biopsies. In the currently reported patient, CMV infection was confirmed by viral culture as well as by polymerase chain reaction.

Severe esophageal candidiasis is usually treated with fluconazole from 100 to 400 mg once daily orally or intravenously for 14 to 21 days but may require therapy extended to 28 days in patients with advanced AIDS [13]. From 50 to 60% of patients with AIDS experience ≥1 recurrent esophageal candidial infection per year despite episodic therapy and require repeat therapy [14]. CMV esophagitis is usually treated with ganciclovir or valganciclovir. Typical initial therapy is intravenous ganciclovir at 10–15 mg/kg/day in 2-3 divided doses/day for 3 to 6 weeks. Maintenance therapy with IV ganciclovir is indicated for GI infection recurrence after discontinuing therapy [15].

## 5. Learning Points

- We report a novel case of massive esophageal candidiasis and simultaneous profound CMV esophageal ulcers in a patient with AIDS.
- Both esophageal infections recurred 12 years later.
- After 12 years, the cytomegaloviral ulcers were so profound as to mimic esophageal pseudo-diverticula on barium esophagram. This case is only the second reported case of cytomegalovirus infection mimicking esophageal pseudo-diverticula. However, the prior case was only reported as an abstract and only had pathology consistent, but not diagnostic, of cytomegaloviral esophagitis.
- In AIDS patients presenting with dysphagia, the differential diagnosis includes simultaneous esophageal candida and cytomegalovirus and considers cytomegalovirus in the differential of esophageal pseudodiverticulosis.
- Patients with other causes of immunocompromise, from chemotherapy, hematologic malignancies, or receiving immunosuppressive drugs after solid organ or bone marrow transplantation, can also rarely develop simultaneous esophageal candidial and CMV infections.
